# Drug treatment patterns in bipolar disorder: analysis of long-term self-reported data

**DOI:** 10.1186/2194-7511-1-5

**Published:** 2013-05-03

**Authors:** Michael Bauer, Tasha Glenn, Martin Alda, Kemal Sagduyu, Wendy Marsh, Paul Grof, Rodrigo Munoz, Emanuel Severus, Philipp Ritter, Peter C Whybrow

**Affiliations:** Department of Psychiatry and Psychotherapy, Universitätsklinikum Carl Gustav Carus, Technische Universität Dresden, Fetscherstr. 74, Dresden, 01307 Germany; ChronoRecord Association Inc., Fullerton, CA 92834 USA; Department of Psychiatry, Dalhousie University, Halifax, NS B3H 4R2 Canada; Department of Psychiatry, University of Missouri Kansas City School of Medicine, Kansas City, MO 64110 USA; Department of Psychiatry, University of Massachusetts, 55 Lake Avenue North, Worcester, MA 01655 USA; Department of Psychiatry, University of Toronto, Toronto, Ontario M5S 1A1 Canada; Mood Disorders Centre of Ottawa, Ottawa, Ontario K1G 4G3 Canada; Department of Psychiatry, University of California San Diego, San Diego, CA 92093 USA; Department of Psychiatry and Biobehavioral Sciences, Semel Institute for Neuroscience and Human Behavior University of California Los Angeles (UCLA), 300 UCLA Medical Plaza, Los Angeles, CA 90095 USA

**Keywords:** Bipolar disorder, Drug treatment patterns, Drug load, Polypharmacy

## Abstract

**Background:**

The objective of this study is to investigate drug treatment patterns in bipolar disorder using daily data from patients who received treatment as usual.

**Methods:**

Patients self-reported the drugs taken daily for about 6 months. Daily drug use and drug combinations were determined for each patient, both by the specific drugs and by medication class. The drug load was calculated for all drugs taken within a medication class.

**Results and discussion:**

Four hundred fifty patients returned a total of 99,895 days of data (mean 222.0 days). The most frequently taken drugs were mood stabilizers. Of the 450 patients, 353 (78.4%) took a stable drug combination for ≥50% of days. The majority of patients were taking polypharmacy, including 75% of those with a stable combination. Only a small number of drugs were commonly taken within each medication class, but there were a large number of unique drug combinations: 52 by medication class and 231 by specific drugs. Eighty percent of patients with a stable combination were taking three or less drugs daily. Patients without a stable combination took drugs but made frequent changes. Taking more than one drug within a medication class greatly increased the drug load.

To summarize, (1) patients were more likely to take a mood stabilizer than any other drug; (2) although most patients were taking polypharmacy, there were no predominant drug regimens even among those taking a stable combination; and (3) most patients with a stable combination take a relatively small number of drugs daily. The wide variation in drug regimens and numerous possible drug combinations suggest that more evidence is needed to optimize treatment of bipolar disorder.

## Background

Drug treatment of bipolar disorder is a difficult challenge due to the recurrent, episodic, and heterogeneous nature of the disease. In recent studies, less than one third of patients in the USA and about half of those in Europe received monotherapy, while the balance received polypharmacy (Azorin et al. 2009; Baldessarini et al. 2008a, b; Goldberg et al. 2009; Hayes et al. 2011; Quante et al. 2010). Many factors have contributed to the increasing use of polypharmacy for bipolar disorder. A substantial proportion of patients do not respond adequately to monotherapy, and additional drugs are required to achieve acute stability and reduce relapses (Goldberg et al. 2009; Frye et al. 2000; Lin et al. 2006; Malhi et al. 2012; Post et al. 2010). There is increased recognition that even while euthymic, patients experience frequent subsyndromal symptoms (Bauer et al. 2010) and an impaired quality of life (Michalak et al. 2005). The drugs available for long-term maintenance have different efficacy for preventing mania and depression, suggesting the need for complimentary agents (Smith et al. 2007). Additionally, with the expansion of drugs available for bipolar disorder such as new-generation antipsychotics and antidepressants, and with psychiatric indications for anticonvulsants, there are numerous choices for the prescriber. Prior research on drug treatment patterns is primarily based on health plan claims (Baldessarini et al. 2007, 2008a) or physician prescribing records (Azorin et al. 2009; Goldberg et al. 2009; Hayes et al. 2011; Quante et al. 2010). This study explored drug treatment patterns in bipolar disorder using 6 months of daily self-reported data on psychotropic drug intake.

## Methods

Data were obtained from an ongoing, long-term naturalistic study in which patients with bipolar disorder recorded mood, sleep, and drugs taken daily (Bauer et al. 2012). All study participants have a diagnosis of bipolar disorder by DSM-IV criteria, are at least 18 years old, receive pharmacological treatment, and agreed to use self-reporting software daily for 6 months. The study has minimal inclusion criteria to better represent the patient heterogeneity of clinical practice. The diagnosis of bipolar disorder was made by the prescribing psychiatrist in a clinical interview. The demographic profile of the patients in this study is similar to those who participated in five large studies of bipolar disorder (Bauer et al. 2012). All patients received pharmaceutical treatment as usual throughout the study. All participants were volunteers and provided written informed consent. The study was approved by each local institutional review board.

### Data collection

Patients self-reported all drugs taken daily, using ChronoRecord software in their native language installed on a home computer. The ChronoRecord software was previously validated and described in detail (Bauer et al. 2004, 2008). In addition to drugs taken, patients enter mood, sleep, and significant life events daily, and weight weekly. During patient training, each drug taken for bipolar disorder was selected from a list of psychotropic drugs in the software, displayed by brand and generic names for the country where the patient resides. For each selected drug, the pill strength was chosen from a list of available strengths. Every day, for each drug, the patient entered the total number of pills taken. Patients could enter partial pills (1/4, 1/2, or 3/4) for tablets but not capsules. If a drug was not taken, the patient entered 0 pills for that drug. The patient could modify the drugs taken throughout the study period as needed, and a drug not included in the software list could be added by the patient.

### Medication classes

Four medication classes were analyzed: mood stabilizers, antidepressants, antipsychotics, and benzodiazepines. Mood stabilizers were defined as lithium, valproate, lamotrigine, carbamazepine, or oxcarbazepine. Second-generation antipsychotics are also effective mood stabilizers, but were classified separately to better distinguish the medications taken and to enable comparison with prior research. Although controlled trials of an antimanic effect for benzodiazepines are lacking (Curtin and Schulz 2004), benzodiazepines were included since they are commonly prescribed to patients with bipolar disorder (Baldessarini et al. 2007; Simon et al. 2004).

### Polypharmacy

Polypharmacy was defined as the use of two or more psychotropic drugs. Polypharmacy may involve two or more drugs from the same medication class such as two mood stabilizers or from different medication classes such as a mood stabilizer and an antidepressant.

### Drug analyses

The daily self-reported data were used for all analyses. For each patient, for each day, taking a drug was defined as taking at least a fraction of a pill per day, regardless of dose. Taking a medication class was defined as taking any drug within the medication class per day. A patient was considered to have a stable drug combination when the same combination of one or more drugs was taken for ≥50% of days. The stable drug combinations were determined to focus the analysis on medications used long term. For all patients, the number of daily changes to the drug combinations was also calculated.

### Drug load

The World Health Organization developed a methodology to calculate drug load that is based on a measure of equipotency known as the defined daily dose (DDD; WHO 2011). The DDD is the average maintenance dose per day for a drug used for its main indication in adults. The ratio of the prescribed daily dose (PDD) to the DDD converts the daily dose strength into multiples of the DDD. A ratio of 1 means the dose taken is equal to the DDD, a ratio greater than 1 means the dose taken is larger than the standard dose, and a dose less than 1 means the dose taken is smaller than the standard dose. The drug load is the sum of the PDD/DDD ratio for all drugs taken within one medication class. Researchers have used a PDD/DDD ratio of 1.5 as the cutoff for an excessive drug load for antipsychotics (Barbui et al. 2006; Procyshyn et al. 2010) and a cutoff of 2.0 for anticonvulsants (Lammers et al. 1995).

The drug load was determined for antidepressants, benzodiazepines, and antipsychotics, but not for anticonvulsants since the available DDD was for the primary indication of epilepsy. In this study, the actual dose taken by the patient was used as the PDD. Only patients who took any drug within a medication class for ≥50% of days were included in the calculation. The PDD/DDD ratio was calculated for each drug and summed for all drugs taken within a medication class. If a patient took multiple regimens with the same drug count within a medication class, the PDD/DDD ratio for each regimen was weighted by the percent of days taken.

### Statistics

Patients were included in this analysis if they returned a minimum of 150 days of drug data, including entries of 0 pills taken. The demographic characteristics of the patients with and without a stable drug combination were compared using chi-square tests for frequency of categorical variables or independent sample *t* tests for mean values of continuous variables. Unequal variance was assumed for all *t* tests. The demographic characteristics of patients with a stable drug combination who took different medication classes were also compared. Means are presented ± the standard deviation (SD). SPSS version 20.0 (IBM SPSS, Armonk, NY, USA) was used for all calculations.

## Results

Data were collected from 513 patients with bipolar disorder, of which 450 (87.7%) returned sufficient data to be included in the study. The 450 patients returned a total of 99,895 days of data (mean 222.0 days). Of the 450 patients, 315 (70%) resided in the USA, 53 (11.8%) resided in Germany, 45 (10%) resided in Canada, and 37 (8.2%) resided elsewhere. The patients had a diagnosis of bipolar I disorder (272, 60%), bipolar II disorder (157, 35%), or bipolar NOS (17, 4%). With a mean age of 40 ± 11.3 years, 313 (70%) were female and 137 (30%) were male. The patients reported 2.4 ± 4.0 lifetime hospitalizations for bipolar disorder and an age of onset of 22.0 ± 10.3 years. For the 450 patients, Table 1 shows the number and percent of days taking any drug within a medication class. Of the 242 patients taking an antipsychotic, only 31 (12.8%) of patients were taking a typical antipsychotic for a total of 2.5% of days. Within each medication class, most of the 450 patients took a small number of specific drugs. As shown in Table 2, only eight antidepressants, five antipsychotics, and three benzodiazepines were taken by at least 5% of the patients.

**Table 1:** **Number of patients taking medication classes by percent of days (** ***N*** **= 450)**

	Mood stabilizers	Antidepressants	Benzodiazepines	Antipsychotics
	***N***(%)	***N***(%)	***N***(%)	***N***(%)
Not taking	63 (14.0)	150 (33.3)	269 (59.8)	208 (46.2)
Taking	387 (86.0)	300 (66.7)	181 (40.2)	242 (53.8)
Total	450 (100.0)	450 (100.0)	450 (100.0)	450 (100.0)
Percent of days taking				
>0 to <10	8 (2.1)	22 (7.3)	38 (21.0)	26 (10.7)
≥10 to <50	20 (5.2)	42 (14.0)	32 (17.7)	40 (16.5)
≥50 to <90	41 (10.6)	36 (12.0)	34 (18.8)	36 (14.9)
≥90	318 (82.2)	200 (66.7)	77 (42.5)	140 (57.9)
Total taking	387 (100.0)	300 (100.0)	181 (100.0)	242 (100.0)

Includes any drug taken within the medication class.

**Table 2:** **Most common drugs taken by medication class (** ***N*** **= 450)**

Medication	***N***(%) Patients
Mood stabilizers	
Lamotrigine	204 (45.3)
Lithium	151 (33.6)
Valproate	107 (23.8)
Oxcarbazepine	51 (11.3)
Carbamazepine	27 (6.0)
Antidepressants	
Bupropion	100 (22.2)
Venlafaxine	53 (11.8)
Escitalopram	44 (9.8)
Sertraline	42 (9.3)
Trazodone	41 (9.1)
Fluoxetine	37 (8.2)
Citalopram	37 (8.2)
Duloxetine	29 (5.4)
Benzodiazepines	
Clonazepam	97 (21.6)
Lorazepam	62 (13.8)
Alprazolam	38 (8.4)
Antipsychotics	
Quetiapine	110 (24.4)
Olanzapine	60 (13.3)
Aripiprazole	53 (11.8)
Risperidone	43 (9.6)
Ziprasidone	30 (6.7)

Drugs taken by 5% or more of the patients.

The drug load analysis revealed that the more drugs taken within a medication class, the larger the mean PDD/DDD ratio (Table 3). Of the 450 patients, 236 were taking an antidepressant for ≥50% of days; 36% of their drug regimens included two or more antidepressants and had a PDD/DDD ratio of >1.5. Of the 450 patients, 176 were taking an antipsychotic for ≥50% of days; 23% of their drug regimens included two or more antipsychotics and had a PDD/DDD ratio of >1.5.

**Table 3:** **Mean PDD/DDD ratio by number of drugs and medication class**

Number of drugs in class	Number of regimens^a^(%)	Mean (SD) PDD/DDD ratio
Antidepressants^b^		
1	204 (64)	1.3 (1.1)
2	97 (30)	2.5 (1.6)
3	15 (5)	3.5 (2.0)
4	3 (1)	5.1 (1.5)
Benzodiazepines^c^		
1	107 (80)	0.6 (0.8)
2	24 (18)	1.4 (1.3)
3	2 (2)	4.0 (1.5)
Antipsychotics^d^		
1	167 (77)	0.7 (0.7)
2	47 (22)	2.2 (2.0)
3	3 (1)	7.4 (6.5)

^a^Some patients took multiple regimens with different number of drugs within a medication class; ^b^236 patients took antidepressants for 50% or more days; ^c^111 patients took benzodiazepines for 50% or more days; ^d^176 patients took antipsychotics for 50% or more days.

Of the 450 patients, 353 (78.4%) took a stable drug combination for ≥50% of the days. There were no significant differences between the demographic characteristics of the patients who did or did not take a stable combination. Considering the stable combinations, there were 52 unique combinations when categorized by medication class (Table 4) and 231 unique combinations when categorized by specific drugs (Table 5). The characteristics of the stable drug combinations are summarized in Table 6. Of the 353 patients with a stable combination, 293 (83%) were taking a mood stabilizer and 82.4% took three or less drugs. The stable drug combinations involved more than one medication class in 248 (70.3%) of the 353 patients. Of the 353 patients, those taking antidepressants were older (42.7 versus 37.2 years, *p* < 0.001) and more likely female (58.5% of females versus 43.8% of males, *p* = 0.010) than those not taking antidepressants. Those taking benzodiazepines were more likely female (27.4% of females versus 12.5% of males, *p* = 0.002) than those not taking benzodiazepines.

**Table 4:** **Most frequent stable combinations by medication class (** ***N*** **= 353)**

Combinations by class	Patients
	***N***(%)
1 Mood stabilizer	62 (18)
1 Mood stabilizer + 1 antidepressant	45 (13)
1 Mood stabilizer + 1 antipsychotic	31 (9)
1 Mood stabilizer + 1 antidepressant + 1 antipsychotic	15 (4)
1 Antidepressant	15 (4)
2 Mood stabilizers	15 (4)
2 Mood stabilizers + 1 antipsychotic	13 (4)
1 Mood stabilizer + 1 antidepressant + 1 benzodiazepine	12 (3)
2 Mood stabilizers + 1 antidepressant	10 (3)
1 Antidepressant + 1 antipsychotic	10 (3)
1 Mood stabilizer + 2 antidepressants + 1 antipsychotic	10 (3)
1 Mood stabilizer + 2 antidepressants	9 (3)
1 Antipsychotic	8 (2)
1 Mood stabilizer + 1 benzodiazepine	7 (2)
2 Mood stabilizers + 1 benzodiazepine	7 (2)
2 Mood stabilizers + 1 antidepressant + 1 antipsychotic	6 (2)
1 Mood stabilizer + 1 antidepressant + 1 benzodiazepine + 1 antipsychotic	5 (1)
2 Antidepressants + 1 antipsychotic	5 (1)
1 Mood stabilizer + 2 antidepressants + 1 benzodiazepine	5 (1)

Only combinations taken by five or more patients included. Data from 292 (83%) of 353 patients and 19 (37%) of 52 unique combinations by class.

**Table 5:** **Most frequent stable combinations by specific drug (** ***N*** **= 353)**

Combinations by drug	Number of patients
Monotherapy	
Lithium	21
Lamotrigine	20
Valproate	17
Bupropion	4
Quetiapine	4
Oxcarbazepine	3
Citalopram	2
Clonazepam	2
Paroxetine	2
Olanzapine	2
Two drugs	
Lithium + lamotrigine	8
Lamotrigine + quetiapine	5
Lamotrigine + venlafaxine	5
Valproate + bupropion	4
Lamotrigine + clonazepam	4
Lithium + quetiapine	4
Valproate + lamotrigine	3
Lamotrigine + bupropion	3
Lamotrigine + citalopram	3
Lamotrigine + risperidone	3
Oxcarbazepine + fluoxetine	3
Oxcarbazepine + quetiapine	3
Bupropion + escitalopram	2
Carbamazepine + citalopram	2
Carbamazepine + lamotrigine	2
Valproate + quetiapine	2
Valproate + sertraline	2
Lamotrigine + fluvoxamine	2
Lamotrigine + escitalopram	2
Lamotrigine + fluoxetine	2
Lithium + venlafaxine	2
Lithium + olanzapine	2
Fluoxetine + olanzapine	2
Oxcarbazepine + bupropion	2
Venlafaxine + aripiprazole	2
Three drugs	
Lamotrigine + clonazepam + quetiapine	2
Lamotrigine + venlafaxine + clonazepam	2
Lithium + carbamazepine + olanzapine	2
Lithium + lamotrigine + clonazepam	2
Lithium + lamotrigine + quetiapine	2
Lithium + lamotrigine + olanzapine	2
Four or more drugs	
Lithium + lamotrigine + clonazepam + quetiapine	2
Lamotrigine + bupropion + paroxetine + quetiapine + aripiprazole	2

Only combinations taken by more than one patient included. Data from 167 (47%) of 353 patients and 43 (19%) of 231 unique combinations by drug.

**Table 6:** **Characteristics of the stable drug combinations (** ***N*** **= 353)**

	***N***(%)
Number of drugs	
1	87 (24.6)
2	117 (33.1)
3	87 (24.6)
≥4	62 (17.6)
Mean number (SD)	2.4 (1.2)
Number of medication classes	
1	105 (29.7)
2	160 (45.3)
3	72 (20.4)
4	16 (4.5)
Mean number (SD)	2.0 (0.8)
Taking medication class	
Mood stabilizer	293 (83.0)
Antidepressant	190 (53.8)
Benzodiazepine	80 (22.7)
Antipsychotic	142 (40.2)

Most of the 97 patients without a stable drug combination were taking drugs but with less consistency. Only 26 patients took no drugs for ≥50% days. Few of the 97 patients took any drug in a medication class for ≥90% of days. Of the 97 patients, 84 (86.6%) were taking a mood stabilizer. Although 47 of the 84 (56%) were taking a mood stabilizer for ≥90% of days, they were not taking the same drug throughout the sample period. Of the 97 patients, 69 (71.1%) were taking an antipsychotic. Of the 69, 14 (20.3%) were taking any antipsychotic for ≥90% of days, but this reflected the sum of days taking different antipsychotics. Patients without a stable drug combination also had twice as many daily changes in drugs taken than those with a stable combination (16 versus 8 changes per year, *p* < 0.001).

## Discussion

Patients were more likely to take a mood stabilizer than any other drug. About 80% of the 450 patients were taking a stable drug combination for 50% or more days. Consistent with prior research (Baldessarini et al. 2008a, b; Goldberg et al. 2009), the majority of patients were taking polypharmacy including 75% of those with a stable combination. About half the patients with a stable combination were taking an antidepressant, despite the ongoing controversy. There was no predominant treatment regimen, as the stable combinations consisted of 52 unique combinations by medication class and 231 unique combinations by specific drugs.

This study cannot distinguish whether or not patients were taking polypharmacy because they were refractory to monotherapy. In a study of US medical claims, about one third of patients with bipolar disorder received initial treatment with polypharmacy (Baldessarini et al. 2008a). Also, polypharmacy is routinely prescribed to inpatients (Greil et al. 2012; Vincenti et al. 2010; Wolfsperger et al. 2007). Recent research suggests that monotherapy with a mood stabilizer or second-generation antipsychotic is generally effective for acute mania (Goldberg et al. 2009; Grunze et al. 2009; Malhi et al. 2012; Tamayo et al. 2010), but polypharmacy is often required for acute depression, mixed states, and maintenance (Goldberg et al. 2009; Malhi et al. 2012; Tamayo et al. 2010). However, polypharmacy increases the risk of drug interactions and adverse events (Besag and Berry 2006; Mojtabai and Olfson 2010; Sandson et al. 2005). In a US national study, psychotropics were a common cause of emergency department visits for adverse drug reactions for those under age 65 (Lucado et al. 2011). The challenge of polypharmacy is that the gain in stability from adding another drug must outweigh the safety risks and side effects (Malhi et al. 2012).

In the current study, the use of more than one drug within a medication class greatly increased the drug load. Patients taking two antipsychotics, antidepressants, or benzodiazepines had about double the drug load of those taking one drug, similar to prior findings (Procyshyn et al. 2010; Tognoni 1999). This result suggests that within a medication class, increasing the dose of one drug produces a lower drug load than adding a second drug. In patients with epilepsy, anticonvulsant polypharmacy is associated with a much higher drug load than with monotherapy, and a higher drug load is associated with increased adverse events (Lammers et al. 1995; Deckers et al. 2001; Deckers 2002). In contrast to that in epilepsy, polypharmacy in bipolar disorder primarily involves drugs from more than one medication class, as with 70% of the patients taking a stable combination in this study. There are few studies of the relation between drug load as measured by the PDD/DDD ratio and adverse events for psychotropic drugs. For example, while persistent exposure to antipsychotic polypharmacy increases the risk of adverse reactions (Carnahan et al. 2006; Centorrino et al. 2004), the optimal measure to predict adverse events is unclear (Barr et al. 2010; Nosè et al. 2008). However, the drug load concept may be useful for improving tolerability of polypharmacy in bipolar disorder, and investigation of the relation between drug load and adverse reactions is indicated.

It is noteworthy that over 80% of the patients with a stable combination were taking three or less drugs. A relatively small number of drugs may facilitate long-term use, and there may be an upper limit on the number of daily drugs beyond which adherence decreases (Bauer et al. 2009; Robertson et al. 2008). Polypharmacy regimens that require frequent dosing, have dietary or time requirements, or are expensive may contribute to nonadherence (Cramer et al. 1989; Ingersoll and Cohen 2008). Patients with bipolar disorder consistently report that side effects contribute to nonadherence (Baldessarini et al. 2008b; Perlick et al. 2004). In this study, the patients without a stable combination were taking drugs, but inconsistently, with drug changes more than once a month. This finding is in agreement with prior evidence that nonadherence in bipolar disorder is typically partial or intermittent rather than complete (Baldessarini et al. 2008a; Lingam and Scott 2002).

There are many limitations to this analysis. All study patients had access to a psychiatrist and were taking medication. Furthermore, the very process of daily recording may improve medication adherence (van Berge Henegouwen et al. 1999). In clinical practice, most patients would not volunteer to keep a long-term daily record. This study may be less generalizable to other patients with bipolar disorder. With a naturalistic design, the patients in this study varied in severity, phase and course of the disease, and years of illness. The study included more females than males and more patients with bipolar I than bipolar II disorder. All data were self-reported and daily access to a computer was required. The strengths of this study include the use of prospective data from a relatively large number of patients and the entry of specific drugs and dose taken daily. However, the prescribed regimens and reasons for addition of new drugs were not known. Some drugs may be misclassified as to prescribing intent. For example, trazodone is considered an antidepressant but is frequently prescribed as a hypnotic agent. The analyses did not include other classes of psychotropic drugs such as stimulants, drugs taken for medical conditions, and over-the-counter preparations. Finally, many diverse factors that may influence medication selection such as physician prescribing habits, drug costs, regulatory influences, insurance plan formularies, pharmaceutical marketing, and patient preferences were not considered (Thistlewaite et al. 2010; Poulsen 1992).

## Conclusions

Treatment of bipolar disorder is individualized in clinical practice as illustrated by 231 unique stable drug combinations for 353 patients. Other than the use of a mood stabilizer, no patterns were found within the stable combinations. A very large number of drug combinations may be prescribed. Considering only the most commonly taken drugs in this study (eight antidepressants, five antipsychotics, and four mood stabilizers), there are 32 possible combinations of mood stabilizer and antidepressant drugs, 20 possible combinations of mood stabilizer and antipsychotic drugs, and 160 possible combinations of mood stabilizer, antidepressant, and antipsychotic drugs. There will never be clinical trials of all possible combination therapies for bipolar disorder. Although controlled studies of polypharmacy are increasing, only limited evidence of effectiveness and safety is available to assist the clinician (Lin et al. 2006; Malhi et al. 2012; Beynon et al. 2009; Ghaemi and Ko, 2002). Further complicating drug selection, patients often have comorbid psychiatric and medical illnesses (Kupfer 2005; McIntyre et al. 2006), including those related to psychotropic drug use (McIntyre et al. 2006; Fagiolini et al. 2005). Some drug regimens in this study may appear inconsistent with current guidelines, but we assume that these are the best regimens for the circumstances of the individual patient. However, the wide variation in drug regimens and numerous possible drug combinations suggests that clinicians need more evidence to optimize treatment. While neuroscience research offers hope for a future with specifically targeted drugs (Insel 2009), the routine use of polypharmacy highlights the importance of clinical judgment in the current treatment of bipolar disorder.
